# Spectral domain optical coherence tomography for quantitative evaluation of drusen and associated structural changes in non-neovascular age-related macular degeneration

**DOI:** 10.1136/bjo.2008.137356

**Published:** 2008-12-03

**Authors:** K Yi, M Mujat, B H Park, W Sun, J W Miller, J M Seddon, L H Young, J F de Boer, T C Chen

**Affiliations:** 1 Harvard Medical School, Boston, Massachusetts, USA; 2 Massachusetts Eye and Ear Infirmary, Glaucoma Service, Boston, Massachusetts, USA; 3 Kangnam Sacred Heart Hospital, Hallym University, Seoul, Korea; 4 Massachusetts General Hospital, Wellman Center for Photomedicine, Boston, Massachusetts, USA; 5 Physical Sciences Inc., Andover, Massachusetts, USA; 6 Boston University, Department of Physics, Boston, Massachusetts, USA; 7 Massachusetts Eye and Ear Infirmary, Retina Service, Boston, Massachusetts, USA; 8 Tufts University School of Medicine, New England Medical Center, Boston, Massachusetts, USA; 9 Department of Physics and Astronomy, VU University, Amsterdam, The Netherlands

## Abstract

**Background/aims:**

To demonstrate how spectral domain optical coherence tomography (SDOCT) can better evaluate drusen and associated anatomical changes in eyes with non-neovascular age-related macular degeneration (AMD) compared with time domain optical coherence tomography (TDOCT).

**Methods:**

Images were obtained from three eyes of three patients with AMD using an experimental SDOCT system. Both a titanium–sapphire (Ti:sapphire) laser and a superluminescent diode (SLD) were used as a broadband light source to achieve cross-sectional images of the retina. A qualitative and quantitative analysis was performed for structural changes associated with non-neovascular AMD. An automated algorithm was developed to analyse drusen area and volume from SDOCT images. TDOCT was performed using the fast macular scan (StratusOCT, Carl Zeiss Meditec, Dublin, California).

**Results:**

SDOCT images can demonstrate structural changes associated with non-neovascular AMD. A new SDOCT algorithm can determine drusen area, drusen volume and proportion of drusen.

**Conclusions:**

With new algorithms to determine drusen area and volume and its unprecedented simultaneous ultra-high speed ultra-high resolution imaging, SDOCT can improve the evaluation of structural abnormalities in non-neovascular AMD.

Since Huang *et al* described the technology of optical coherence tomography (OCT) over 15 years ago,^1^ OCT has become a widely used technology in ophthalmology. The leading commercially available instrument, the StratusOCT (Carl Zeiss Meditec, Dublin, California), is based on time domain OCT (TDOCT) technology. With the StratusOCT, two-dimensional cross-sectional retinal images consisting of 512 A-lines with axial resolutions of 10 μm can be obtained in 1.28 s. In order to improve resolution, ultra-high resolution (UHR) OCT imaging, which also utilises time domain technology, was developed and could achieve axial resolutions of about 3 μm.^2^ ^3^ UHR OCT imaging, however, requires longer acquisition times, and two-dimensional images consisting of 3000 axial and 600 transverse pixels would take several seconds to obtain.^4^

In spectral domain optical coherence tomography (SDOCT) technology, the light from the reference arm interferes with the light reflected back from the different layers of the retina, generating spectral interference fringes. This fringe pattern is processed by a high-speed spectrometer, and then undergoes Fourier transformation to create a reflectivity profile in depth. Therefore, SDOCT has also been called Fourier domain OCT (FDOCT).^5^ The three-dimensional structure of the retina can therefore be reconstructed by laterally scanning the laser beam across the retina. Instead of acquiring depth information by looking at the change in interference pattern in time, as in TDOCT, the SDOCT system acquires depth information by analysing the interference pattern in the spectrum of mixed reflected lights.

SDOCT’s fundamentally different detection method which utilises a spectrometer is more efficient and therefore allows for a 150-fold improvement in sensitivity compared with equivalent TDOCT systems.^6^^–^9 This higher sensitivity allows for faster acquisition speeds and for detection of weaker signals.^10^ Therefore, axial resolutions of about 2 μm are possible with SDOCT.^11^ Ultrahigh speeds of 34.1 μs per A-line can be obtained. Single images comprising 1000 A-lines can be acquired in 34.1 ms or 1/29 of a second. These faster acquisition speeds allow SDOCT to scan larger areas of the retina.

Our study seeks to demonstrate how SDOCT may improve the clinical care of AMD patients, in that our new algorithm can determine drusen area and volume automatically. Past studies have shown that drusen diameter and area may be significantly associated with risk of progression to advanced AMD over 5 years; however, the detailed analysis of drusen diameter and area made it cumbersome to use clinically.^16^^–^18 With this new algorithm, SDOCT technology could potentially provide more objective and easier classification of AMD according to drusen size or area, which will help determine the disease prognosis for certain patients.

In this paper, we demonstrate SDOCT images of three eyes of three patients with non-neovascular AMD. We demonstrate a new automated algorithm which calculates drusen area and volume.

## METHODS

Study protocols were approved by the Massachusetts Eye and Ear Infirmary and Massachusetts General Hospital Institutional Review Boards and in accordance with the Health Insurance Portability and Accountability Act. Patients were enrolled after informed consent was obtained.

An experimental SDOCT instrument was developed at the Massachusetts General Hospital, Wellman Center for Photomedicine. The descriptions of the setup and the basic theory have been published in detail.^9^ ^10^ ^12^ For the light source, both a superluminescent diode (SLD, Superlum, Russia) with a full width at half maximum (FWHM) spectral width of 50 nm centred at 840 nm and a Ti:Sapphire laser with a FWHM spectral width of 140 nm centred at 800 nm were used. The incident optical power in the eye was about 600 μW in both cases, well below the American National Standards Institute (ANSI) standards.^13^ A retinal tracker as described in previous publications was utilised.^14^ ^15^ The purpose of the lateral retinal tracker was to compensate for involuntary eye motions during the scan that would manifest as image discontinuities and jumps that are typically seen in StratusOCT images.

The axial distance was calibrated by measuring a mirror in a model eye with 100 μm incremental steps by translating a mirror in the reference arm, giving peaks in the depth profiles at 100 μm intervals. The resulting measured distance was divided by 1.38 to correct for the average refractive index of the retinal tissue.^9^ The lateral distance was calibrated by measuring the angular sweep entering the eye. The angle was converted to a distance assuming a fixed eye length of 23 mm and a vitreal refractive index of 1.34, giving a conversion factor of ∼300 μm/°. Refractive error does not affect the lateral distance calibration, but an eye length that deviates from an average of 23 mm may. We assume an average eye length of 23 mm, since we excluded high myopes from our study, and we only included patients with refractive errors of −5 dioptres to +5 dioptres.

**Figure 1:**
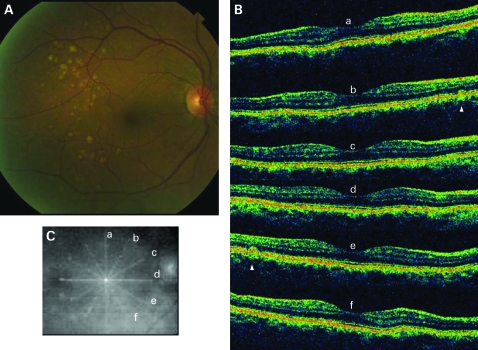
Colour fundus photograph and StratusOCT fast macular scan of the right eye of a patient with dry age-related macular degeneration (patient A). (A) Multiple large soft drusen temporal to the macula. (B) Six standard OCT radial scans showing only two frames show small drusen (arrowhead). (C) Radial lines indicating the positions and directions of the six radial scans.

Images from patients A and B are shown to compare information received from StratusOCT versus SDOCT imaging (fig 1). Images from patients B and C are shown to demonstrate pigmentary clumping, atrophy and drusen in non-neovascular AMD.

**Figure 2:**
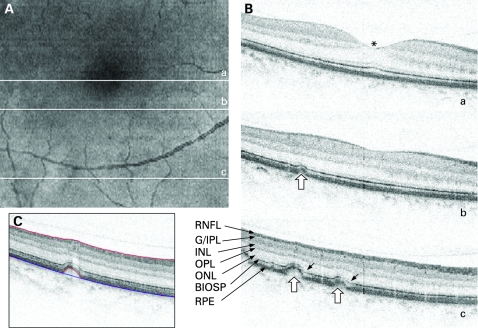
Spectral domain optical coherence tomography (SDOCT) images of the right eye of a patient with dry age-related macular degeneration (patient A). (A) Integrated reflectance image. Letters a, b and c indicate the position of the selected frames. (B) Upper frame: centre of the fovea (a, asterisk, top right); middle frame: from inferior to the fovea (b, middle right). Last frame (c, bottom right): several drusen temporal to macula (hollow arrows). Note the reduced reflectance of the boundary between the inner and outer segments of the photoreceptors (IS/OS) around drusen (short arrows). (C) Baseline (blue line) and drusen boundary (lower red line) as determined by our algorithm. The single frames are expanded vertically by 2.67 for better visualisation of the retinal layers. The light source was a Ti:sapphire laser with axial resolution of 3 μm. G/IPL, ganglion cell/inner plexiform layer; INL, inner nuclear layer; IS/OS, boundary between the inner and outer segments of the photoreceptors; ONL, outer nuclear layer; OPL, outer plexiform layer; RNFL, retinal nerve fibre layer; RPE, retinal pigment epithelium.

A quantitative analysis of SDOCT images from patient A was performed (figs 2^3^). The automatic algorithm^6^ searched along each depth profile (A-line) for the lowest boundar, y in the OCT image which corresponded to the posterior retinal pigment epithelium (RPE) border. In areas where drusen were detected, the identified RPE boundary was determined to be the anterior boundary of the drusen. To determine the posterior border of the drusen (blue line, fig 2C), the original RPE boundary was then fitted with a second-order polynomial to determine the original RPE position (baseline) before the drusen accumulation occurred. The difference between the identified posterior RPE border and calculated baseline provided the geometric dimensions of the drusen (lower red line, fig 2C). The dimensions of drusen were determined based on the fundus photographs, too.

**Figure 3:**
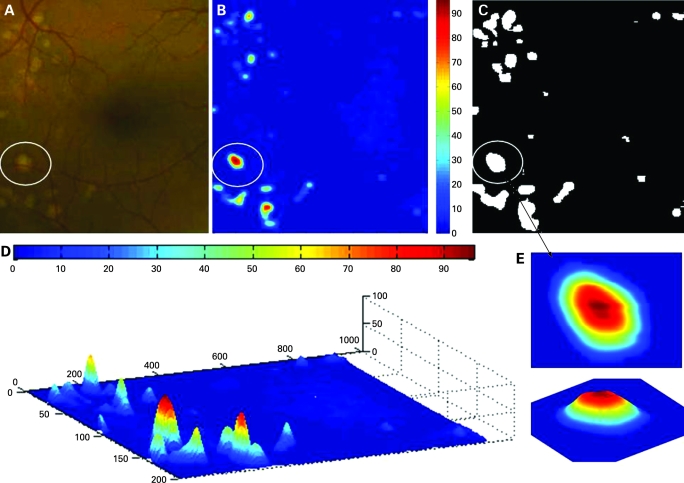
Results of the automated drusen analysis from fig 2. (A) Retinal area of drusen analysis. (B) Corresponding retina with a false-colour scale after analysis. (C) Retina area with drusen, elevated above the baseline more than 8 µm (5 pixels of 1.6 µm), shown as white. The proportion of retina with drusen was calculated as 5.85% of the total scan surface. (D) Analysed retina displayed three-dimensionally. The height was elongated in this figure by five times. The colour bars are scaled as actual size in microns. (E) One of the drusen (circled, from top figures) shown without height elongation. The calculated area of this druse is 1.46×10^5^ μm^2^ or 0.146 mm^2^, and the volume is 9.31×10^6^ μm^3^ or 9.31×10^−3^ mm^3^. The total area of scan was 4.97 mm×5.18 mm×1.24 mm.

Time domain optical coherence tomography images were obtained using the StratusOCT (Carl Zeiss Meditec). The fast macular thickness map protocol was used.

Patients were recruited from the Massachusetts Eye and Ear Infirmary and were as follows:

- Patient A: The right eye of a 54-year-old black female was imaged with both StratusOCT and SDOCT. She was noted to have multiple large drusen on a routine eye examination and was diagnosed as having non-neovascular AMD. Her corrected visual acuity was 20/20 OD with −3.00 −0.25×75°.
- Patient B: The right eye of an 82-year-old Caucasian male was imaged with both StratusOCT and SDOCT. He had initially been diagnosed as having non-neovascular AMD 14 years ago. There were multiple large drusen in his retina. His best corrected visual acuity was 20/60 OD with +0.50 −2.50×105°.
- Patient C: The right eye of a 58-year-old Caucasian male was imaged with SDOCT. He had several retinal pigmentary clumps in his macula and was diagnosed as having non-neovascular AMD. The visual acuity of that eye was 20/25 with +1.50 −0.25×70°.

Prior to imaging, eyes were dilated with 5% phenylephrine hydrochloride and 0.8% tropicamide.

## RESULTS

The right eye of patient A showed multiple soft drusen temporal to the macula (fig 1A). A StratusOCT macular scan only showed drusen in two of the six radial scans (fig 1B,C, white arrowheads). Drusen or retinal pigment epithelium (RPE) elevations could not be found easily on the other radial scans (a, c, d, f). Patient A was also imaged with SDOCT (fig 2). These pictures were select frames from a 4.97 mm×5.18 mm×1.24 mm volume scan, which consisted of 200 frames acquired at 29 frames per second. The original frames were made of 1000 A-lines, but they were cropped to 960 A-lines per frame to remove side artefacts due to fast scanning. On these SDOCT images, multiple elevations consistent with drusen are clearly shown (hollow arrows).

**Figure 4:**
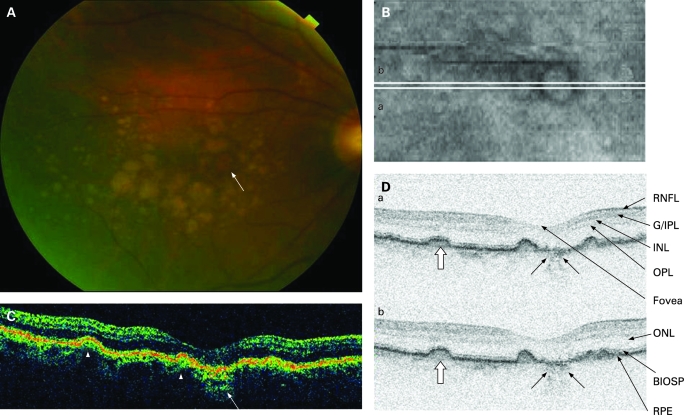
Colour fundus photograph and optical coherence tomography (OCT) imaging of the right eye of a patient with dry age-related macular degeneration (patient B). (A) Small degree of geographic atrophy noted nasal to the fovea (short arrow). (B) Integrated reflectance image. Letters a and b indicate the position of the selected frames. (C) Horizontal scan from the StratusOCT showing multiple drusen (arrowheads). (D) Spectral domain OCT images. Increased transmission due to pigment loss (short arrows) is noted along with the drusen (arrowheads). The light source was a Ti:sapphire laser with an axial resolution of 3 μm. G/IPL, ganglion cell/inner plexiform layer; INL, inner nuclear layer; IS/OS, boundary between the inner and outer segments of the photoreceptors; ONL, outer nuclear layer; OPL, outer plexiform layer; RNFL, retinal nerve fibre layer; RPE, retinal pigment epithelium.

**Figure 5:**
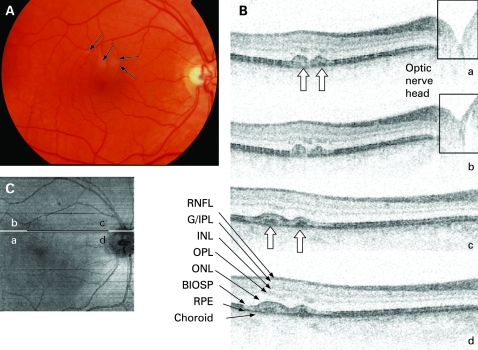
Colour fundus photograph and spectral domain optical coherence (SDOCT) images of the right eye of a patient with dry age-related macular degeneration (patient C). (A) Several pigment clumpings with surrounding atrophy (arrows) in the superior macula in fundus photography. (B) Small hyper-reflective elevations (hollow arrows) just above the level of the retinal pigment epithelium (RPE) corresponding to the pigment clumping noted on the colour fundus photo. Frames a and b were expanded vertically by 2.7, and frames c and d by 1.5 for better visualisation of the retinal layers. The light source was a superluminescent diode laser with an axial resolution of 6 μm. A retinal tracker was applied. G/IPL, ganglion cell/inner plexiform layer, between the inner and outer segments of the photoreceptors; RNFL, retinal nerve fibre layer; RPE, retinal pigment epithelium. (C) Integrated reflectance image. The letters a, b, c and d indicate the position and width of the selected frames.

The black and white fundus images shown in the left panel of fig 2, right panel of fig 4 and left panel of fig 5 were obtained from the three-dimensional SDOCT scans by simply integrating each reflectivity depth profile (or A-line). These images, called integrated reflectance images, are very similar to scanning laser ophthalmoscope (SLO) images.^6^ Although both SLO and OCT use scanning laser beams, a typical SLO uses a single detector that collects the light reflected from all the retinal layers measuring an overall reflectance. By integrating the reflectivity profile in OCT, we can obtain the same overall reflectance. The additional depth discrimination of OCT distinguishes these two technologies; however, for the purpose of illustrating the en face retinal map, the two technologies can display similar images. These SDOCT integrated reflectance images can generate fundus images with a similar resolution and quality to that of SLO. The horizontal lines across these SDOCT integrated reflectance images indicate the position of the two-dimensional scans shown either below or to the right side of these figures.

For patient A, the posterior border of the RPE was analysed three-dimensionally (figs 2, 3). For a quantitative analysis of drusen, the identified RPE boundary from the automated algorithm^6^ was fitted with the calculated original RPE position (baseline; fig 2C). The difference between these two curves delimited the final drusen dimensions.

Figure 3 shows the result of the drusen analysis algorithm, which calculates the drusen area (fig 3B,C), shape (fig 3D), and volume (fig 3E). The colour bars are scaled in micrometres and denote the height of the drusen. For example, the calculated base area of a single druse (fig 3E) is 0.146 mm^2^, and the volume of this drusen is 9.31×10^−3^ mm^3^.

The dimension of the drusen can be mapped with false-colour coding (fig 3B). A two-dimensional binary map of drusen within the retinal SDOCT scan area (fig 3C) shows the drusen shape at the baseline, as obtained from an analysis of the SDOCT data. Drusen which are elevated above the baseline more than 8 µm (5 pixels of 1.6 µm) are shown as white, and the proportion of retina with drusen was calculated as 5.85% of the total scan surface.

Patient B (fig 4) had multiple large drusen. StratusOCT scans show multiple drusen (fig 4C, arrowheads). The RPE irregularity is partly caused by motion artefact and may make it difficult to distinguish a small druse from motion artefacts. The SDOCT scan shows the striking features of multiple discrete round RPE elevations (fig 4D, arrowheads). Geographic atrophy (maximum linear dimension of 567 μm) is noted nasal to the fovea on the fundus photo (fig 4A, short arrow), which corresponds to the Straus OCT images (fig 4C, short arrows) and SDOCT images (fig 4D, short arrows), both of which show increased transmission due to pigment loss. This geographic atrophy fits into the category of central geographic atrophy, with a grade 3 size for the Age-related Eye Disease Study (AREDS) grading scale.^16^ The scanned volume is 5.0 mm×5.18 mm×1.56 mm. One hundred frames were acquired at 29 frames per second, with 963 A-lines displayed for each frame. The RPE is straighter and with less motion artefact compared with the StratusOCT images.

Patient C had several areas of pigment clumping with surrounding atrophy in the superior macula (fig 5A, arrows). One of these pigment clumps was sized as 223 μm, which fitted into grade 4 of the AREDS grading scales (ie, increased pigment size equal to or above 125 μm but less than 250 μm).^16^ From his SDOCT scans, four frames are shown (fig 5B). Frames a and b are SDOCT images from a 6.35 mm×6.9 mm×1.67 mm scanned volume. Frames c and d are SDOCT images from a 3.81 mm×6.9 mm×1.67 mm scanned volume. Both settings have 460 A-lines for one frame, with 150 frames acquired at 29 frames per second. Frames from both of the settings scanned a similar area of retina (shown as the white and black horizontal lines on the integrated reflectance image). For better delineation of structures, frames are expanded vertically by 2.7 times for frames a and b but by only 1.5 times for frames c and d. Small hyper-reflective elevations (hollow arrows) just above the level of the RPE correspond to the pigment clumping noted in fig 5 and imply RPE hyperplasia. The overlying retinal layers look well preserved.

## DISCUSSION

OCT is a valuable imaging tool for the evaluation of retinal diseases, because it is non-invasive and relatively affordable. Most importantly, OCT provides ophthalmologists with clinically relevant images that correlate well with histology.^10^ ^19^ ^20^ Numerous advances in the diagnosis of retinal diseases as well as improved understanding of retinal pathology can be obtained with OCT technology, and OCT is now also used to guide therapy.^21^

The leading commercially available OCT instrument (StratusOCT) as well as the experimental UHR systems have utilised time domain technology. SDOCT can now allow for unprecedented simultaneous ultra-high speed ultra-high resolution ophthalmic imaging.^9^ ^10^ SDOCT can allow for scanning of the entire posterior pole of the retina, instead of just scanning the macula with six radial scans and using interpolation to account for missing data between the radial scans as is currently used for the fast macular scan in the StratusOCT instrument. StratusOCT detected only two drusen (fig 1) compared with the numerous drusen actually seen on fundus photography and SDOCT scanning (figs 2, 3).

SDOCT images also have fewer motion artefacts than StratusOCT. In fig 4, some areas of RPE between the drusen show undulation due to motion artefact in the StratusOCT image. In the SDOCT image for the same patient (fig 4D), the flatter RPE and retinal layers are relatively free from motion artefact, and the margins of the drusen are clearer. This shows how SDOCT can improve the ease with which we can interpret OCT images.

Because of its faster acquisition speeds, SDOCT can provide much more information compared with the standard TDOCT (fig 3). For example, SDOCT can create three-dimensional scans of large areas (ie, up to a 30° field of view) in under 10 s and can also display three-dimensional tomographic videos of the optic nerve head and retina. In contrast, the slow acquisition speed of TDOCT can only practically scan two-dimensional images and does not have video capabilities. SDOCT’s more comprehensive visualisation of retinal structures could potentially improve the evaluation and treatment of AMD. Because SDOCT also allows for higher-resolution imaging, this imaging technology may further decrease the need for repeated fluorescein angiography.

By the end of 2006, some companies obtained FDA approval to market SDOCT machines. Some of these companies include Heidelberg Engineering (Spectralis, Germany), Carl Zeiss Meditec (Cirrus, Dublin, California), Optopol Technology Sp. z.o.o. (SOCT Copernicus, Zawiercie, Poland), and Optovue Co. (RTVue-100, Fremont, California).

Interestingly, there is reduced reflectance of the boundary between the inner and outer segments of the photoreceptors (IS/OS) around drusen (fig 2, short arrows). This reduced reflectance is not seen with the lower-resolution StratusOCT images (figs 1, 4). Even with cases of non-neovascular AMD, the photoreceptor layer, especially rods, can be damaged because of the dysfunctional RPE.^22^ This may be associated with structural changes in the photoreceptor layer and the IS/OS that may result in reduced reflectivity of the IS/OS, especially in areas overlying drusen. The elevation of RPE over the dome-shaped drusen may also change the reflectance of the IS/OS by disrupting the original orderly vertical arrangement of photoreceptors. These are both possible explanations for the decreased reflectance of the IS/OS.

New automated algorithms that determine the total volume or area of all scanned drusen as well as the proportion of retinal area with drusen could potentially provide meaningful parameters for evaluating AMD disease level and progression (fig 3). Drusen area as well as drusen size has been proven as an important indicator of AMD progression.^16^^–^18 On a recent report describing a simplified severity scale, AREDS implied that drusen size was used instead of drusen area, because assessment of drusen area was more difficult.^17^ SDOCT may solve the problem of drusen assessment (fig 3). These algorithms may also facilitate treatment decisions. For example, according to the AREDS, drusen size may be a parameter used to decide whether a patient would benefit from AREDS vitamin supplements.^23^ An automated algorithm that determines drusen area objectively would potentially make the decision whether to use AREDS vitamin supplements quicker for the treating physician. Reproducibility and validation studies of this algorithm are still needed.
